# Electrical storm in the inflamed heart: ventricular tachycardia due to myocarditis

**DOI:** 10.1002/ccr3.1071

**Published:** 2017-07-03

**Authors:** Gustav Mattsson, Peter Magnusson

**Affiliations:** ^1^ Centre for Research and Development Uppsala University/Region Gävleborg Gävle SE‐ 801 87 Sweden; ^2^ Cardiology Research Unit Department of Medicine Karolinska Institutet Stockholm SE‐171 76 Sweden

**Keywords:** Antiarrhythmic, cardiac magnetic resonance, electrical storm, implantable cardioverter defibrillator, myocarditis, risk stratification, sudden cardiac death, ventricular tachycardia

## Abstract

Electrical storm during the acute inflammatory phase caused by myocarditis may be resistant to antiarrhythmic therapy. Cardiac imaging including magnetic resonance tomography, positron emission tomography, and endomyocardial biopsy are crucial to guide potential therapeutic options. Optimal management involves a multidisciplinary approach, including expertise beyond cardiology.

## Introduction

Myocarditis is an inflammation of the heart muscle, with widely varying etiology and severity. In the Western world, it is believed to be most commonly caused by a virus, but bacteria, fungi, parasites, or protozoa are also known causes of infectious myocarditis. Moreover, there are immune‐mediated causes (e.g., systemic lupus erythematosus, Churg‐Strauss syndrome, sarcoidosis, or Wegener's granulomatosis), toxic causes (e.g., drugs, heavy metals, and snake or scorpion venom), and damage from ionizing radiation or electricity 1.

Symptoms of myocarditis include palpitations, chest pain/discomfort, acute onset of or sudden worsening of heart failure (HF), and cardiac arrhythmias. Cardiac troponins are usually elevated. ECG abnormalities range from unspecific abnormal Q waves or ST inversions to diffuse concave ST segment elevations without reciprocal changes 2, 3.

Typically, myocarditis has a mild course without the need for advanced medical management; as such, it is often unrecognized. However, life‐threatening arrhythmias have been described in the setting of myocarditis. We here present a case of myocarditis causing an aggressive, long‐lasting, electrical storm (ES) with several diagnostic and therapeutic challenges.

## Case Report

A 49‐year‐old woman presented at the emergency department with chest discomfort, dizziness, fatigue, and pallor. She was treated solely with duloxetine for depression and anxiety. Three years earlier, she had presented with HF, elevated troponin levels, and a normal coronary angiogram and was diagnosed as having takotsubo cardiomyopathy.

**Figure 1:**
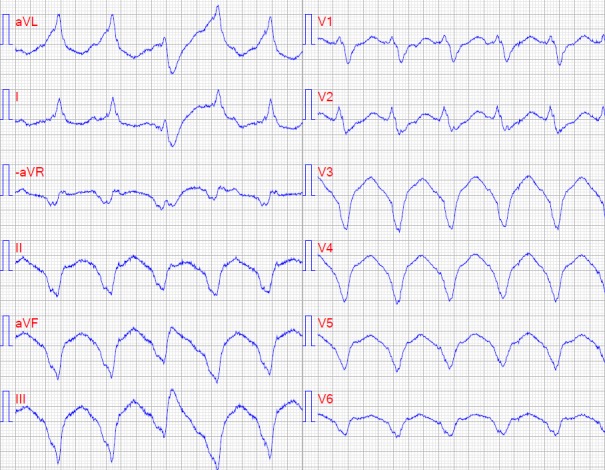
ECG showing VT (170 bpm) with suspected right apical ventricular origin.

At arrival, she was alternating between a sinus rhythm of 80 beats per minute (bpm) and a monomorphic ventricular tachycardia (VT) of 170 bpm with hemodynamic compromise but no syncope. The ECG revealed VT of suspected right ventricular apical origin (Fig. 1). Troponin T was 221 *μ*g/mL at admission. The recurring VT was treated with metoprolol and a bolus of 300 mg of intravenous (IV) amiodarone and the rhythm converted into sinus rhythm. An echocardiogram showed apical and septal hypokinesia, minimal mitral insufficiency, and a left ventricular systolic ejection fraction (EF) estimated at 35%. Two hours after admission to the hospital, the VT returned, but could be aborted after another bolus of IV amiodarone followed by a potassium and magnesium infusion.

**Figure 2:**
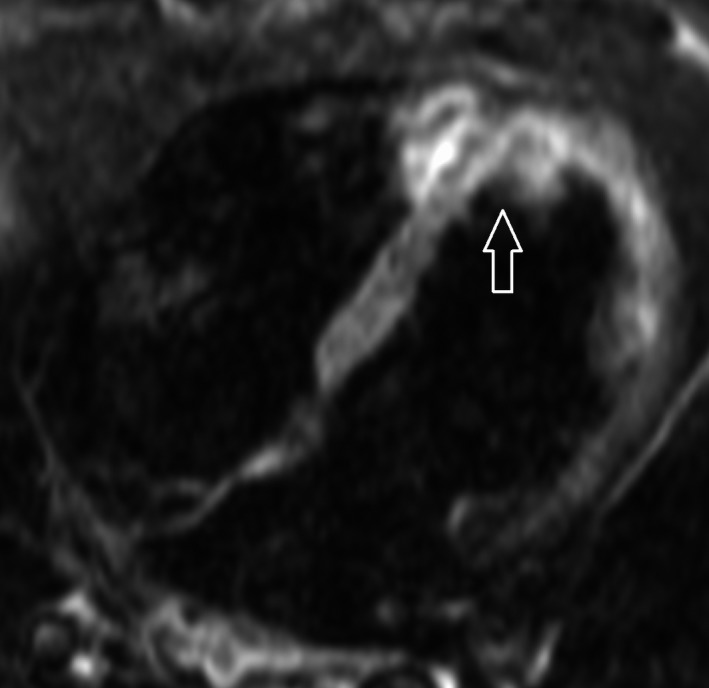
Four‐chamber view, T2‐weighted image: signs of edema (arrow) in the apical septum and left ventricular wall.

**Figure 3:**
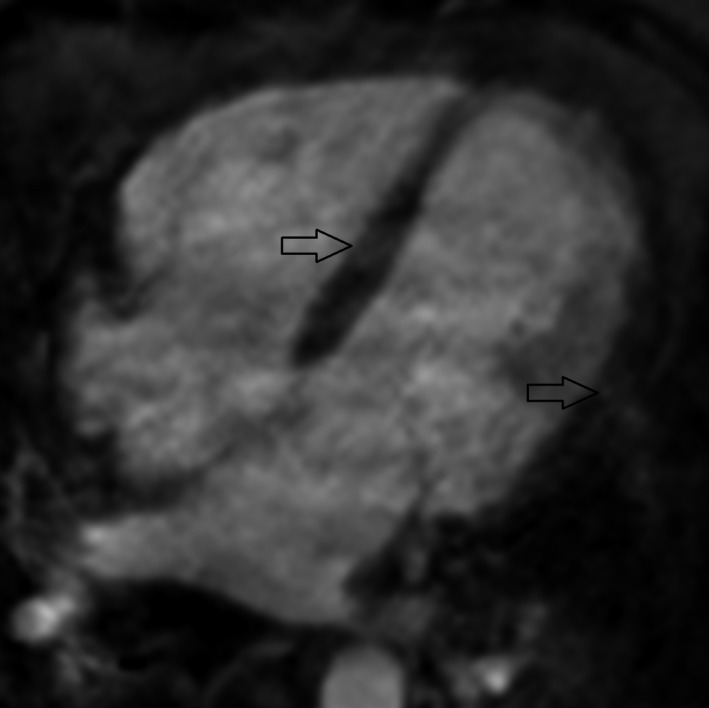
Four‐chamber view, early contrast: early gadolinium enhancement (arrows) in the septum and left ventricular wall.

**Figure 4:**
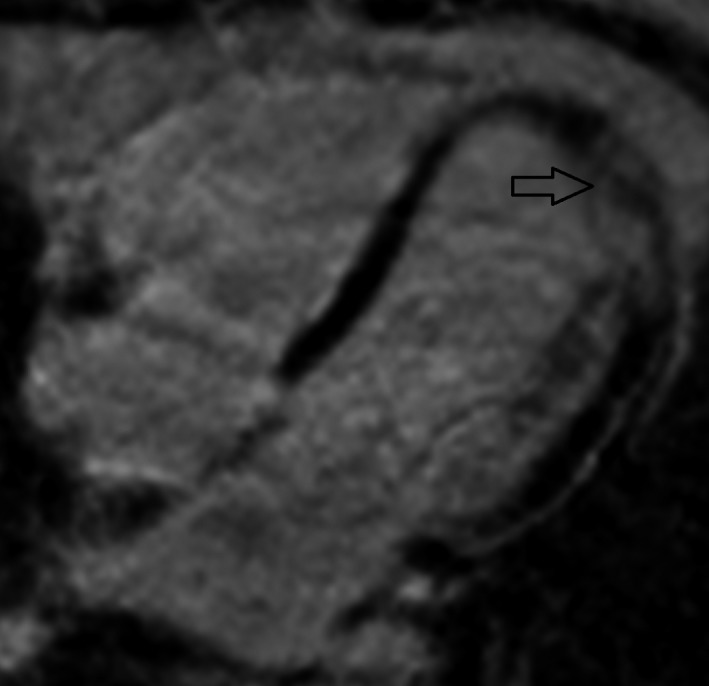
Four‐chamber view, late contrast: late gadolinium enhancement (arrow) in the apical part of the left ventricle.

**Figure 5:**
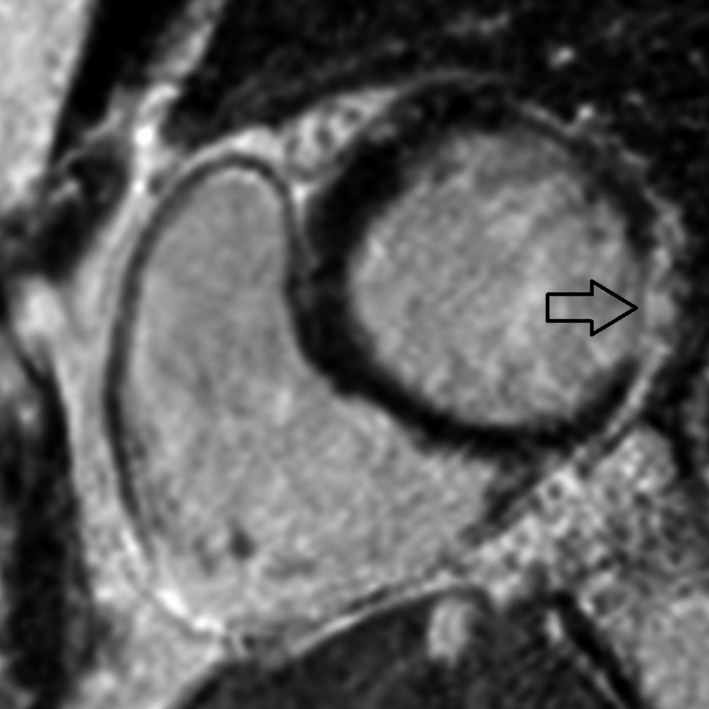
Short‐axis view, late contrast: late gadolinium enhancement (arrow) in the left ventricular wall and in the septum. Enhancement is patchy and multifocal with tendency toward endocardial sparing.

The patient was transferred for an angiogram to exclude coronary artery disease as the culprit for the arrhythmia. The angiogram was normal and recurrent VT every hour required repeated boluses and continuous administration (1200 mg during 24 h) of IV amiodarone. HF medications, including metoprolol and enalapril, were initiated. A cardiac magnetic resonance (CMR) tomography scan showed signs of inflammation and edema, dilation of the left and right ventricles, with EF 29% and 27%, respectively (Figs 2, 3, 4, 5). Cardiac sarcoidosis was suspected based on patchy areas of late gadolinium enhancement and 30 mg prednisolone daily was started. Despite supranormal dosages of amiodarone, VT recurred and the treatment strategy needed to be changed. The ES did not cease (up to 40 min of VT) even by the third day, and lidocaine (100 mg bolus followed by continuous infusion of 2 mg/min) was administered despite the patient's depressed biventricular function. The recurrent VT became worse, however, and a higher heart rate (190 bpm) complicated the situation. She was referred to the thoracic intensive care unit at a tertiary center.

Her pharmacological regimen was revised: antiarrhythmic therapy was switched back to IV amiodarone, lidocaine was discontinued, and spironolactone was added to treat her underlying HF. Midazolam and dexmedetomidine were used for sedation to decrease sympathetic tone. Notably, the patient's C‐reactive protein increased to 190 mg/L from 3.7 mg/L 3 days earlier; her sedation rate was at 90 mm/h in the absence of focal symptoms of infection, and cefotaxime and doxycycline were given. VT may cause hemodynamic instability, so the patient was sedated with propofol and intubated. Over the next 24 h, she was cardioverted several times. After several cardioversions in the following days, the situation stabilized, and amiodarone was changed to an oral regimen.

Blood and urine cultures were negative. Viral and bacterial serology was negative, except for elevated IgM for *Borrelia burgdorferi* deemed not indicative of current infection. ANCA and serum angiotensin‐converting enzyme levels were normal. Interestingly, there was a family history of rheumatic disease; two aunts and her father had rheumatoid arthritis; it had also been suspected that her father had microscopic polyangiitis, a condition confirmed in the patient's daughter. The unspecific marker ANA was elevated, but without clinical signs of systemic lupus erythematosus. Corticosteroids were discontinued after a positron emission tomography–computer tomography (PET‐CT) showed receding inflammation and no signs of sarcoidosis. After 18 days, C‐reactive protein levels were close to normal and VT was infrequent, so ablation was deemed unnecessary. Furthermore, the VT had several ECG morphologies, suggesting complex ablation procedure, which was a reason to postpone ablation and wait until the inflammation had receded. Endomyocardial biopsy (EMB) was not performed. After 25 days, she experienced another VT, was sedated with propofol, and reverted to sinus rhythm before cardioversion could be performed. The following day, an implantable cardioverter defibrillator (ICD) with a single lead was implanted as her QRS < 120 msec. After a total of 30 days in the hospital, she was discharged, and at her three‐month follow‐up, no VT was seen on device interrogation and amiodarone was discontinued after another month.

## Discussion

Electrical storm is usually defined as three or more separate episodes of sustained VT within 24 h, is often life‐threatening, and has been associated with poor prognosis 4. The underlying etiology varies and it is important to consider triggers: electrolyte disturbances, proarrhythmic drugs, acute coronary syndrome, and structural heart disease 5. Electrolytes should be corrected and potassium levels between 3.5 and 4.5 mmol/L have been associated with a lower risk of VT in ischemia 6. While magnesium is considered beneficial in polymorphic VT, specifically *torsades de pointes* 7, it was administered to this patient as well despite the fact that she had monomorphic VT. It is advisable to scrutinize the medication history, including dosage, with regard to potential proarrhythmic properties; fluoxetine was discontinued for this reason.

In sustained VT with hemodynamic instability, prompt cardioversion is needed. With hemodynamically stable VT, cardioversion is a first‐line therapy, while IV amiodarone and beta‐blockers (and possibly flecainide or verapamil) may be considered 5. In our case of recurrent VT, beta‐blockers in combination with amiodarone were initially effective, but later general anesthesia and cardioversion were necessary. General anesthesia and sedation might reduce the recurrence of VT by reducing the sympathetic tone 5, 8.

Amiodarone may be used for prophylaxis in recurrent monomorphic VT 5, 8 and lidocaine, considered only moderately effective, may be a short‐term option 5. Flecainide is contraindicated in ischemic heart disease, HF, and its use as an IV regimen is not readily available in Sweden 9. The sodium channel blocker lidocaine should be used with caution and under careful monitoring for EF depression, as was done in this case. When lidocaine turned out to be ineffective in calming the storm, the decision was made to switch back to amiodarone. In accordance with guidelines, the patient was transferred to a unit able to perform emergency electrophysiological catheter ablation. However, the situation stabilized after the patient was sedated and her myocarditis ran its natural course. Furthermore, ablation is not without risks and it is not clear in this case how effective it would be, as the patient's arrhythmia had different ECG morphologies, which may reflect a complex substrate or multiple foci, due to the underlying inflammatory process 10. A 12‐lead ECG can provide information on the mechanism and origin of VT in ES. Repeated echocardiograms are needed to monitor HF and NT‐proBNP is a sensitive marker in HF. Because ischemic heart disease is common, it is crucial to rule it out as the cause of ES and angiography is recommended early in the management of ES 5, 11.

Myocarditis should be suspected considering the patient's symptoms, the presence of ES, and her new‐onset HF 1, 2. CMR imaging could be performed to diagnose myocarditis and visualize scars. The Lake Louise criteria are used to diagnose myocarditis with CMR 12. In the setting of clinically suspected myocarditis, two of three criteria are necessary for CMR findings to be considered consistent with myocarditis:

- high myocardial signal intensity on T2‐weighted images, indicating edema
- increased early gadolinium enhancement ratio between myocardial and skeletal muscle, indicating vasodilation and increased blood flow
- increased late gadolinium enhancement in at least one focal area of the myocardium, indicating scar tissue retaining contrast.

Our patient fulfilled all three criteria. Patchy, multifocal areas of late gadolinium enhancement with endocardial sparing are commonly seen in sarcoidosis, which led to our suspicion of sarcoidosis and the resulting initiation of corticosteroid treatment 13. The use of corticosteroids in cardiac sarcoidosis is controversial, and optimal dosage and timing with regard to ICD implant are unknown 13, 14. Serum angiotensin‐converting enzyme levels were normal; however, this is the case in up to 40% of patients with sarcoidosis 15. PET‐CT with fluorodeoxyglucose (FDG) is a useful method for diagnosing sarcoidosis 13, 14. There were no signs of sarcoidosis on the PET‐CT, which instead led to a diagnosis of myocarditis of unknown origin. Several laboratory tests to ascertain infective or autoimmune origin were inconclusive. HF due to myocarditis should be treated according to guidelines, including beta‐blockers, angiotensin‐converting enzyme inhibitors or angiotensin receptor inhibitors, and selective aldosterone receptor antagonists 1.

Endomyocardial biopsy is the gold standard for diagnosing myocarditis 1. Biopsy also allows for the use of reverse transcriptase–polymerase chain reaction to detect virus RNA, thus allowing the option of specific antiviral therapy 16. In the absence of a virus, this could also strengthen the suspicion of autoimmune origin, thereby allowing for corticosteroid therapy or other immunosuppressive treatments. The histological picture of inflammation (e.g., lymphocytic or giant cell) affects prognosis and treatment. For example, in giant cell myocarditis, immunosuppression could be considered 17. The value of EMB and the fact that CMR should not replace EMB are pointed out in the current position paper 1. As EMB was not performed in our patient, the optimal treatment and prognosis remain unclear.

After the ES receded, further risk stratification with regard to sudden cardiac death was warranted. If the cause of her VT was transient and HF resolved, the indication for a transvenous ICD could be questioned. During the time of potential recovery, a wearable cardioverter defibrillator might have been suitable 18, 19, 20. Because this patient was young, experienced ES over an extended period of time, and suffered from a reduced EF despite HF medication, it was decided to offer her an ICD. Furthermore, her previous diagnosis of takotsubo was likely myocarditis. This strengthens her ICD indication, in that it means her ES was not transient and, instead, is based on permanent scar‐related substrates. The decision to implant a single‐lead ICD rather than a dual‐chamber system was based on the fact that she had no bradycardia indication. Due to the history of monomorphic VT, a transvenous ICD is preferred over a subcutaneous ICD to offer antitachycardia pacing 21.

## Authorship

GM: involved in the writing of the article, design, and conceptualization. PM: involved in writing of the article, patient management, design, and conceptualization. Both authors: approved the final version of the case report for submission to the Clinical Case Reports.

## Conflict of Interest

None declared.
